# Intracardiac echocardiography is a promising strategy for guiding closure of the left atrial appendage

**DOI:** 10.1002/hsr2.1762

**Published:** 2023-12-18

**Authors:** Xueyan Ding, Kefa Xiang, Congli Qian, Xu Hou, Feng Wu

**Affiliations:** ^1^ Department of Cardiology Sir Run Run Shaw Hospital Zhejiang University School of Medicine Hangzhou Zhejiang P.R. China; ^2^ Department of Cardiology, The 72nd Group Army Hospital Huzhou University Huzhou Zhejiang P.R. China; ^3^ Bengbu Medical College Bengbu Anhui P.R. China

**Keywords:** atrial fibrillation, intracardiac echocardiography, left atrial appendage closure

## Abstract

**Background and Aims:**

Percutaneous transcatheter left atrial appendage (LAA) closure (LAAC) is an effective approach for preventing ischemic stroke in nonvalvular atrial fibrillation patients. Intracardiac echocardiography (ICE), a new imaging modality, is a promising strategy for guiding LAAC. This review highlights the various strategies for ICE‐guided‐LAAC as an option for clinical policy.

**Methods:**

A comprehensive literature search was conducted of PubMed, ScienceDirect, Ovid Web of Science, SpringerLink, and other notable databases to identify recent peer‐reviewed clinical trials, reviews, and research articles related to ICE and its application in the guidance of LAAC.

**Results:**

Various methods are used to evaluate the spatial structure and dimensions of the LAA. The main techniques for guiding LAAC are transesophageal echocardiography (TEE), cardiac computed tomography (CTA), and ICE. Among these techniques, the advantages of ICE typically include (1) multiangle and real‐time assessment of intracardiac structure, (2) a reduction in procedural fluoroscopy, (3) reduced operation time and improved workflow in the catheterization laboratory, and (4) the avoidance of general anesthesia and the early detection of complications.

**Conclusion:**

ICE is a promising strategy for the guidance of LAAC. Among the most advanced and recent technological innovations in cardiovascular imaging in general and volume imaging in particular, ICE offers greater efficacy and safety.

## 1 INTRODUCTION

**Figure 1:**
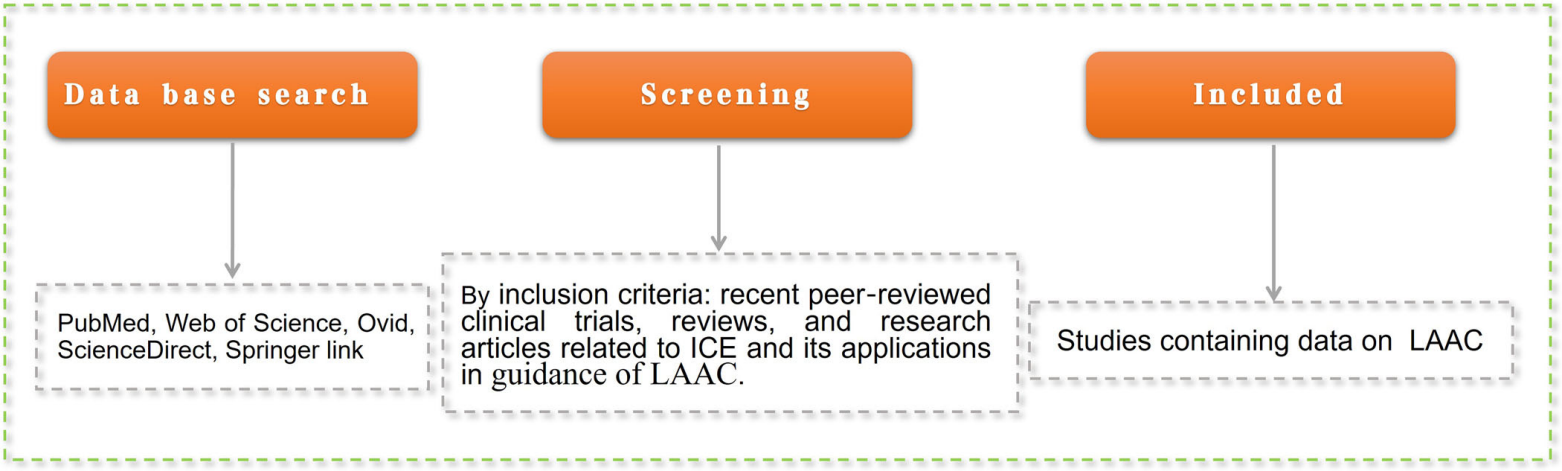
The flowchart of search strategy steps. After searching PubMed, Web of Science, Ovid, ScienceDirect, SpringerLink, and other notable databases, studies were screened by two reviewers. Then, the steps of selecting studies based on the main criteria were done by one reviewer. LAAC, left atrial appendage closure.

As one of the most common types of arrhythmia, atrial fibrillation (AF) increases the risk of ischemic cerebrovascular incidents,^1^ accounting for 30% of strokes in individuals over 80 years of age and 15% of strokes across age groups.^2^ Oral anticoagulants (OACs), which are recommended for patients with CHA2DS2‐VAS scores of ≥2 (males) or ≥3 (females), have proved to be highly effective in reducing the risk of thromboembolism in patients with AF.^3^ Though OACs are a frontline therapy for preventing stroke in patients with AF, their application in some patients with this form of arrhythmia has certain risks and limitations as the existence of relative or absolute contraindications.^4^ Further, previous studies have shown that more than 90% of strokes start with left atrial appendage (LAA) thrombi.^5^ For these reasons, LAA closure (LAAC) has emerged as an effective therapeutic approach for preventing ischemic stroke (class IIb, level B) and is recommended under the current relevant guidelines.^6^ The standard intraoperative guidance for transcatheter LAAC involves various techniques and systems, among which transesophageal echocardiography (TEE) is currently the preference for LAAC guidance. However, general endotracheal anesthesia is required to avoid patients' discomfort and motion during TEE‐guided LAAC.^7^ In an important breakthrough, a minimally invasive approach for LAAC guided by intracardiac echocardiography (ICE) under moderate sedation has been developed.^8^ Since TEE remains the main intraoperative formation of image mode for LAAC in most centers, the guidance of LAAC by ICE is increasingly popular and may become the gold standard. In this paper, we review the various strategies for using ICE‐guided LAAC (Figure 1) and discuss its advantages as an option for clinical policy.

## 2 OVERVIEW OF ICE

### 2.1 Background of ICE

ICE is a unique imaging technique that allows for the immediate visualization of the structure of the heart and display of the adjacent relationships of various parts of the cardiac system and, accordingly, has been adopted as an essential tool for electrophysiology (EP) procedures and percutaneous interventions.^9^, ^10^ Because it provides near‐field, high‐quality, real‐time assessment of cardiac anatomy,^11^ ICE has gradually come to be used to guide cardiac interventional therapy and guard against complications. ICE‐guided cardiac imaging modalities usually involve placing a catheter in the right ventricle (RV), right atrium (RA), or left atrium (LA).^12^ Two other unusual catheter‐placement techniques, coronary sinus echocardiography (CSE) and percutaneous intrapericardial echocardiography (IPE), are rapidly being developed and will have a tiny space in the area of EP. CSE involves the use of catheter‐based ultrasound, specifically, the introduction of an ICE catheter into the coronary sinus (CS), while, in IPE, an ICE catheter is introduced into the pericardial space.^13^, ^14^

More than 60 years ago, researchers first used a catheter with an ultrasonic transducer on dogs to obtain endocardium echo image information about the ventricles by entering the heart cavity through the jugular vein.^15^ Ever since, engineers and scientists have continuously been making breakthroughs and exploiting ultrasonic probes to obtain images of inner cardiac structures.^16^ In the present century, interventional catheterization has become the preferred strategy for diagnosing and treating partial arrhythmias and structural heart disease. As an important interventional auxiliary approach, ICE was initially applied to guide the interventional occlusion of patent foramen ovale and atrial septal defects, its image quality being equivalent to that of TEE.^11^, ^17^ With the continuous improvements in the equipment, such as phased array ultrasound transducers, ICE could meet the complex needs of intraoperative imaging.^18^, ^19^ Currently, though fluoroscopy and electroanatomical mapping remain the primary clinical techniques for catheter navigation in interventional therapy, ICE is increasingly used for this purpose because of the advantages of real‐time monitoring of complications and well‐tolerated procedures.^17^, ^20^ With the domain of cardiovascular interventions continuing to forge ahead, ICE is expected to be an important adjunct for percutaneous interventional and EP procedures.

### 2.2 Types of ICE systems

Currently, two main types of ICE systems are used in clinical practice, radial or rotary ICE and phased‐array ICE.^21^ The radial rotary system available in clinical practice, Ultra ICE™ (Boston Scientific), involves the application of a single piezoelectric crystal affixed to the pointed end of a French catheter with a mechanical 360° rotary ultrasound transducer. Its maneuverability is limited by the rotary ICE catheter (including a shortage of Doppler imaging and catheter diversion),^22^ which must be placed in the RA with the guidance of a long sheath.^23^ Therefore, the radial ICE system is currently used mainly in electrophysiological studies.^24^ Phased‐array ICE, on the other hand, involves the use of many rows of knobs to operate the catheter, with the capacity to flex and fix in multiple directions so as to create two‐dimensional (2D) images, and thus resembles transesophageal and transthoracic echocardiography.^25^ The phased‐array systems for ICE presently available include the ViewFlex™ Xtra ICE catheter (Abbott Vascular), SoundStar ICE catheter (Johnson & Johnson‐Biosense Webster), and AcuNav Volume ICE catheter (Siemens). These systems have a range of 5–10 MHz ultrasound frequency and a 15–16 cm penetration depth and are equipped with a 64‐unit phased‐array ultrasonic transducer at the tip that can have Doppler capabilities and provide greater organizational penetration.^22^

## 3 THE MAIN LAAC GUIDING TECHNIQUES

**Figure 2:**
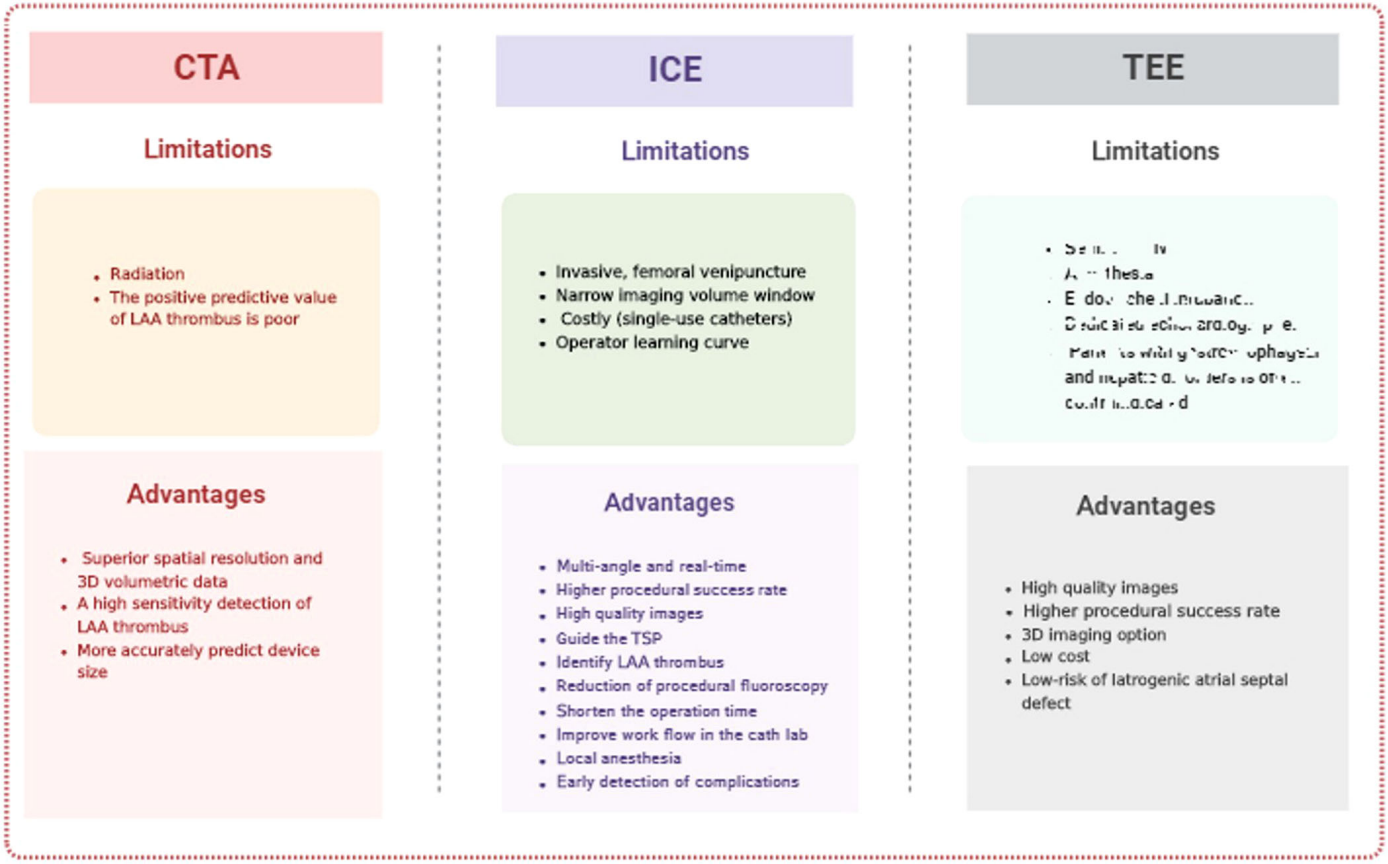
The comparison of different guidance techniques in left atrial appendage occlusion. CTA, cardiac computed tomography; ICE, intracardiac echocardiography; LAA, left atrial appendage; TEE, transesophageal echocardiography; TSP, trans‐septal puncture.

Various methods are used to evaluate the spatial structure and dimensions of the LAA. The main techniques involving guided LAAC include TEE, cardiac computed tomography (CTA), and ICE. Practitioners choose one or another technique based on their expertise, experience, and training. The various guidance techniques for LAAC have distinct limitations and advantages (Figure 2).

### 3.1 Cardiac computer tomography angiography (CCTA)

As one of the most accurate imaging methods, CT has high spatial and temporal resolution, which can overall assess the complicated surrounding and anatomy structures of LAA.^26^ International specialists have reached a consensus on the use of cardiac CT for preprocedural LAAC imaging planning.^27^ Its preoperative use can reduce surgical times and the complications associated with LAAC.^28^ Multidetector cardiac CT can serve to evaluate the LAA and provide for accurate device selection,^29^, ^30^, ^31^ especially for measuring the maximum LAA orifice.^32^ Numerous researchers have confirmed that three‐dimensional (3D) CCTA can predict the dimensions of the device more accurately than fluoroscopy, even TEE.^32^, ^33^ CCTA‐guided LAAC can improve the accuracy of equipment selection and program efficiency and reduce the procedure time and the number of occludes consumed during surgery.^28^ Further, CCTA has high sensitivity in detecting LAA thrombosis, with a negative predictive value of 96%–100%, advising that patients without filling coloboma on CCTA imagings do not require TEE. However, a recent meta‐analysis indicated that the specificity of CCTA for LAA thrombus detection ranged from a low positive predictive value of only 41%–92%.^34^ The filling effect may be caused by an impaired contrast mixture that resembles a thrombus in the LAA and accounts for the poor positive predictive value. The results of a recent study indicate that dual‐energy CCTA helps to distinguish iodine‐enhancing phenomena from nonenhancing lesions through rapid kilovoltage‐switching.^35^ In general, CCTA is gradually coming into use for preoperative preparation and follow‐through.^36^, ^37^ Many clinical studies have investigated fusion CT‐guided LAAC, but they are subject to limitations, and additional technological breakthroughs are needed.

### 3.2 TEE

Currently, TEE is the imaging approach most widely accepted for LAAC. This method offers high specificity and sensitivity (98% and 92%, respectively) as well as high positive and negative predictive values (86% and 100%, respectively) in detecting LAA thrombosis.^38^, ^39^, ^40^ TEE is also typically used to rule out device embolization and pericardial effusion.^41^ Transthoracic echocardiography is necessary to evaluate the anatomical structure and function of the heart in patients preparing for LAAC.^42^, ^43^ In step with the rapid growth of 3D technology in recent decades, 3D TEE has become widely available for calculating the dimensions of the LAA and the volume‐exported ejection fractions.^44^, ^45^ So far, despite the potential risks associated with TEE‐guided LAAC, TEE remains the leading method of intraoperative imaging during the procedure in most centers.^46^

### 3.3 ICE

The first reports of the use of LAAC guided by ICE appeared in 2007.^47^ In 2010, MacDonald et al.^48^ published the first report of the use of ICE as an independent imaging method to guide LAAC by positioning the probe in the pulmonary artery. In 2014, Berti et al.^49^ reported the results of a double‐center study of LAAC guided separately by ICE located in the CS or the RA. In 2016, Frangieh et al.^50^ reported a failure rate of zero among 32 patients when pushing ICE probes into the LA through a single interval puncture during LAAC guidance. However, ICE, as an emerging guidance tool, has been applied optionally in a small number of patients under the operators' careful consideration, and the majority of LAAC still prioritizes TEE guidance.^51^ As a result, the data on the use of ICE in LAAC procedures remain limited. More recently, numerous studies have demonstrated the feasibility, safety, and efficacy of LAAC guided by ICE, and more and more research is confirming that ICE‐guided LAAC using second‐generation occlude devices is safe and effective.^52^, ^53^ Going forward, two prospective studies (ICE WATCHMAN study [NCT04569734] and the ICELAA Clinical Study [NCT04196335]) are underway to validate ICE device release criteria and provide a standardized imaging technique.^54^

## 4 STRATEGIES FOR THE USE OF ICE‐GUIDED LAAC

**Figure 3:**
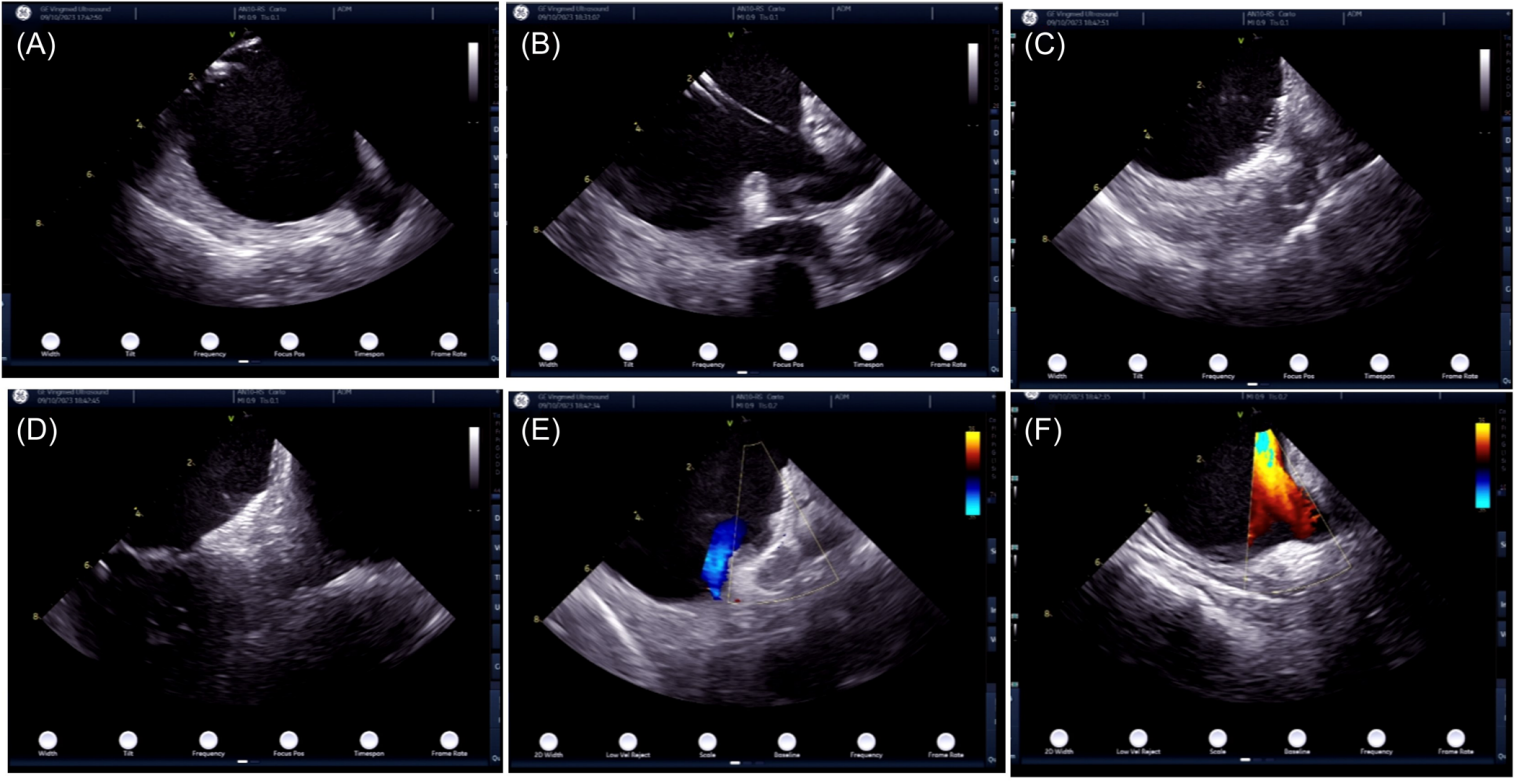
The images of the echo windows in intracardiac echocardiography. (A) Right atrium; (B) right ventricle; (C) left atrium; (D) relationship between left atrium and mitral valve; (E and F) intracardiac blood flow.

ICE can initially image the RA long axis, RV, and tricuspid valve with the insertion of the catheter from a neutral position into the RA. Mitral valve imaging can be provided by rotating the ICE catheter slightly clockwise from a neighbor view and then visualizing the LAA by adjusting the knobs that govern the catheter's front and rear orientation (Figure 3).^55^ The use of ICE from the RA to guide LAAC has also been reported.^56^ An ICE catheter inserted into the RA usually cannot fully display the spatial structure of the LAA, especially in patients with an enlarged LAA or thick fossa ovalis. ICE is also insufficient for the evaluation of LAA thrombosis. However, though the visualization and evaluation of LA from the RA using ICE is less than ideal, the success rate of ICE from the RA guided by LAAC technology reached 97.5% in one study when performed by well‐experienced operators.^49^ Other sites for observing the LAA from the right heart using an ICE catheter include the CS and pulmonary artery, but further investigation of the placement of the catheter in these sites has proved challenging.^41^ Imaging from the pulmonary artery can provide effective observation of the LAA as its anatomy is close, but it must be performed gently to reduce the risk of perforation. When the aortic valve is visible, the catheter is inserted into the left pulmonary artery, and the catheter also provides an excellent LAA image for evaluating thrombus.^57^ Satisfactory images can be obtained from the standard RA position, and better images can be obtained from the ICE view of the CS.^55^ Thus, in a small‐sample study of 10 patients, ICE imaging was optimal during percutaneous LAAC when the ICE probe was located in the CS.^47^ Additionally, it is difficult to visualize the LAA from the right side to assess leakage around the device, so ICE may not be the ideal choice for LAAC.^58^ The optimal LAA visualization can be achieved by placing the ICE catheter at the center of the LA or, in rare cases, at the inflow of the mitral valve.^59^ Alkhouli et al. elaborated on a simplified dual‐view approach that provides a long‐axis view and a short‐axis view from the intracardiac ICE catheter and eliminates the need to advance the catheter into the pulmonary vein, with the device‐release being based only on this dual view. These researchers argued that their approach has clear safety advantages and may present fewer technical challenges for less‐experienced users of ICE.^60^ Korsholm et al. described a three‐view approach of ICE‐guided LAAC that includes a mid‐LA long‐axis view, an left superior pulmonary vein view, and a supra‐mitral view. The instrument‐release criteria are determined based on three or more views, one of which must be from the supra‐mitral visual angle. With the combination of the preprocessed dural CT and the improved design of the Watchman FLX device, the success rate of ICE‐guided implantation for the first time is 96%.^53^ Using three views from the LA, including the main view of mid‐LA long‐axis and a supra‐mitral view, with the ICE imaging plane facing up the LA. When the ICE‐guided LAAC placement method is first used, the author suggests achieving preoperative CT scans of LA and LAA for the initial patient, so that the operator can have a clear idea about the anatomical structure of LAA and become proficient in the 3D navigation of the ICE catheter in LA.^61^

## 5 COMPARISON OF ICE‐GUIDED LAAC AND TEE

The main difference between ICE and TEE is the position of the probe. The TEE probe placed in the esophagus serves to view the rear‐LA anatomy, and the ICE probe is usually delivered through the femoral vein to allow for direct observation of the target anatomy from the cardiac chamber or great vessels.^62^ Several reports have contrasted the roles of ICE and TEE in LAAC. Hemam et al.^51^ compared the effectiveness of ICE‐guided and TEE‐guided Watchman planting strategies and observed a 100% success rate for the implantation in both groups with no differences in terms of perioperative complications or significant postoperative leakage. A multicenter study in Italy compared ICE guidance and TEE guidance for the implantation of Amplatzer devices and observed similar surgical success rates and safety in both modalities.^63^ A subsequent study of 286 consecutive enrolled patients, of whom 196 received TEE and 90 received ICE, found likewise that both groups had similarly high implant success rates (>95%) and a low incidence of complications (<4%).^60^ Furthermore, a meta‐analysis of Amulet and Watchman implants guided by ICE and TEE detected no differences with respect to the acute surgical success rate, surgical time, or complications (e.g., tamponade, instrument embolization, or stroke).^64^ TEE, then, remains the gold standard for LAAC procedure, but there are drawbacks, such as the need for additional anesthesiologists and cardiologists to perform it. By comparison, ICE has certain development advantages and has frequently been used for LAAC surgical guidance as a substitute for imaging, especially in patients with absolute contraindications for TEE or tracheal intubation.

## 6 BENEFITS OF ICE‐GUIDED LAAC

### 6.1 Multiangle and real‐time assessment of intracardiac structure

The LAA is an important part of the heart. A comprehensive 3D evaluation of the LAA is necessary to carry out interventions successfully. Obtaining the diameter and effective working depth of the opening area of the LAA in advance can facilitate the accommodation of the individualized anatomical structures and axial directions of distinctive LAA.^23^ The location, stability, compression, and outcome of the LAA closure equipment can be evaluated in three dimensions after the release of the equipment. Notably, multiangle evaluation is a prominent feature of ICE‐guided LAAC. The progressive nature of ICE imaging is reflected in its value as an electrolytic profiling technical drawing tool for assessing tissue heterogeneity and thickness.^65^ ICE provides accurate real‐time information about tissue thickness that is difficult to obtain, even using preoperative CT or magnetic resonance imaging of LAAC. Inserting an ICE catheter into the LA allows for multiangle scanning around the LAA without the limitations on the use of TEE in patients with cardiac insufficiency and LAA mutations. In particular, ICE facilitates observation of structural transformations and hydroncus formation, thereby increasing the efficacy and safety of LAAC. In addition, the ICE probe can accurately determine whether the LAA has thrombosis before trans‐septal puncture (TSP)^66^, ^67^ and provides a useful perspective for imaging to assess intracardiac thrombus during the LAAC procedure.^57^ Therefore, real‐time imaging is among the prominent benefits of ICE‐guided LAAC.

### 6.2 Reduction of procedural fluoroscopy

To facilitate the acquisition of coaxiality between the drug delivery sheath and the LAA and, thereby, the arrangement of the instruments and the adjustment of their positions, the lower posterior TSP is usually used during LAAC. When variation is observed in the LAA involving inverted wings or proximal downward curvature, median/anterior TSP is recommended.^68^ At most centers, the guidance of TSP routinely depends on TEE and fluoroscopy. However, at a few centers with experienced clinicians, X‐ray fluoroscopy alone is a sufficient guide for TSP and LAAC. A 2009 study by Ferguson et al.^69^ demonstrated the safety and feasibility of TSP guided by ICE under continuous pressure monitoring. Minimizing radiation during surgery has always been a fundamental principle of interventional therapy, and numerous studies have confirmed that ICE can reduce radiation during LAAC. Hemam et al.^51^ reported that the fluoroscopy time of LAAC under ICE guidance decreased by almost one‐third. Kim et al.^70^ reported an approximate 25% reduction in the total radiation from ICE‐guided LAAC compared with TEE‐guided LAAC. Korsholm et al. demonstrated that the fluoroscopy time for the ICE group was comparable to that of the TEE group, but the amount of contrast agent used in the ICE group was significantly less than that used in the TEE group during LAAC.^60^, ^71^ However, these researchers also found no statistically significant difference between the two groups in terms of perspective time and the use of contrast media, a result that may be related to variation in the technical level and experience of the clinicians at the centers. According to recent studies, through the comprehensive evaluation of LAA, combined with the flexible operation of the ICE catheter, LAAC under the 3D electroanatomical mapping system can even usher in a leap of zero contrast agent and zero radiation.^72^ Multiple studies have confirmed that the combination of ICE and 3D‐electroanatomic mapping systems represents a crucial step toward the elimination of radiation from the LAAC procedure and green EP generally.^72^, ^73^, ^74^

Nondose‐related random effects are of greatest concern regarding the risks that radiation poses to human health, especially radiation‐induced carcinogenesis and genetic defects.^75^ The reduction or elimination of fluoroscopy in interventional procedures to reduce the risk of radiation exposure to patients and staff is a serious joint clinical problem.^76^ Over the past decade, the use of fluoroscopy in interventional therapy has been declining thanks to a combination of efforts, including the application of ICE imaging.^69^

### 6.3 Shortening operation time and improving workflow in the catheterization laboratory

ICE has greatly improved the workflow of catheterization laboratories. During TEE‐guided LAAC, a single case requires at least three operators (an interventional physician, an anesthesiologist, and an echocardiography physician), but only one operator is required for ICE‐guided LAAC. In particular, the fact that the latter procedure under local anesthesia requires neither tracheal intubation nor resuscitation after anesthesia optimizes the turnover in the catheter room.^54^ Thus, in a study of 104 patients with AF by Hemam et al.^51^ in which equal numbers of patients received ICE guidance and TEE guidance for LAAC, the operation times were similar for the two groups but the catheterization time for the ICE group as 18% less than that of the TEE group, and the turnaround time was 45% less. Other studies have reported similar findings. Alkhouli et al.^60^ found that, compared with the TEE group, the ICE group in their study had approximately 45% less indoor time. A small‐sample retrospective study by Korsholm et al.^71^ found that the operative, turnaround, and total room times were shorter for the ICE group (*n* = 109) than the TEE group (*n* = 107). Further, in a comparison of the procedural time for LAAC conducted by two groups of operators, one using ICE and the other using TEE, the procedural time for the former group was approximately half that for the former, and a decrease in fluoroscopy times was observed.^77^ Accordingly, as operators become experienced and skilled in the use of ICE technology, the procedural time for ICE‐guided LAAC may decrease further.

### 6.4 Avoidance of general anesthesia and early detection of complications

Patients with AF who undergo LAAC are typically frail older individuals with a high burden of comorbidities and thus stand to benefit from avoiding general anesthesia.^78^ Since surface echocardiography cannot guide LAAC, the fact that ICE does not require general anesthesia is a unique benefit of it as an alternative to TEE for such individuals. ICE can, likewise, be used for patients with liver and gastroesophageal diseases, who typically do not tolerate OAC and for whom TEE is contraindicated. Pericardial effusion/tamponade is a concern for hypotension or hemodynamic impairment as a serious complication of LAAC. ICE can serve to evaluate any baseline effusion through clockwise rotation of the catheter for observation of the left and right ventricles.^10^ An ICE catheter can also detect pericardial effusion in a timely manner, before hemodynamic changes, thereby reducing the risk of death through a modest intervention.

## 7 CURRENT STATUS OF AND FUTURE DIRECTIONS FOR ICE‐GUIDED LAAC

Currently, ICE catheters provide only 2D single‐plane imaging. For assessing the size of the LAA and choosing the appropriate size of instrument, the ICE catheters in most centers are inferior to 3D imaging. Fortunately, 3D ICE probes currently under development^79^, ^80^ are likely to overcome the limitations of 2D ICE. Thus, a recent study found that RT3D ICE can provide immediate detailed feedback on intracardiac structures for a more comprehensive anatomical evaluation during LAAC.^81^

Given the urgent need for advances in ICE imaging, four‐dimensional (4D) ICE holds great promise for LAAC in terms of broadening the imaging field. Real‐time and 3D volume acquisition permit 4D volume ICE technology to depress the demand to capture images from multiple anatomical locations. The capacity to image complex substrates dynamically is expected to increase the efficiency of the guidance of the process.^82^ An innovative recent 4D ICE approach provides a multiplanar reformatted echo view with an increased imaging volume of 90° × 50° for procedural guidance.^83^ The use of 4D ICE imaging from the right heart transcends the necessary premise of providing intraoperative imaging guidance through the septal channel during LAAC.^83^ More clinical studies are needed to demonstrate the superiority of 4D ICE‐guided LAAC with regard to the procedural success rates, standardized workflow, cost‐effectiveness, and compensating for nondiagnostic image quality in cases of challenging patient characteristics.

## 8 CONCLUSIONS

Over the past few decades, LAAC has emerged as an effective nonpharmacological therapy for the prevention of cerebral apoplexy in nonvalvular AF. An essential feature of this new technology is its high‐quality imaging modes, which provide accurate anatomical information during the LAAC procedure. ICE allows for real‐time visualization of the location and occlusion of the LAA and the dynamic display of the spatial relationship between the catheter and key anatomical structures. Further advances in ICE imaging technology are likely to play pivotal roles in the treatment of structural heart disease and in driving innovations in electrophysiological technology.

## AUTHOR CONTRIBUTIONS

**Xueyan Ding**: Writing—original draft. **Kefa Xiang**: Writing—original draft. **Congli Qian**: Writing—original draft. **Xu Hou**: Investigation. **Feng Wu**: Funding acquisition; writing—review & editing.

## CONFLICT OF INTEREST STATEMENT

The authors declare no conflict of interest.

## TRANSPARENCY STATEMENT

The lead author Xueyan Ding affirms that this manuscript is an honest, accurate, and transparent account of the study being reported; that no important aspects of the study have been omitted; and that any discrepancies from the study as planned (and, if relevant, registered) have been explained.

## Data Availability

Data derived from public domain resources.
